# Multiplex Polymerase Chain Reaction (PCR) and Conventional Methods for Diagnosing Ventilator-Associated Pneumonia in ICU Settings: A Systematic Review

**DOI:** 10.7759/cureus.94852

**Published:** 2025-10-18

**Authors:** Aalaa Salih Fadel Yosif, Gidaa Khalid Eltayeb Blado, Mohamed Awad Bashir Eltayeb, Nada Abdelrahman Shamina, Mohamed Abass Ahmed Abdalaziz, Amina Osman

**Affiliations:** 1 Nephrology, Sligo University Hospital, Sligo, IRL; 2 Acute Medicine, Yeovil District Hospital, Yeovil, GBR; 3 Internal Medicine, Northampton General Hospital, Northampton, GBR; 4 Chemical Pathology, Nottingham City Hospital, Nottingham, GBR; 5 Internal Medicine, Luton and Dunstable Hospital, Luton, GBR; 6 Acute Medicine, Queen Elizabeth Hospital, Gateshead, GBR

**Keywords:** antimicrobial stewardship, intensive care unit, molecular diagnostics, multiplex pcr, systematic review, ventilator-associated pneumonia

## Abstract

Ventilator-associated pneumonia (VAP) is a prevalent and serious infection in intensive care units (ICUs), with timely and accurate diagnosis being crucial for patient outcomes. Conventional diagnostic methods, primarily culture-based, are hampered by long turnaround times and limited sensitivity. Multiplex polymerase chain reaction (PCR) (mPCR) offers rapid detection of multiple pathogens and resistance genes, potentially revolutionizing VAP diagnosis and antimicrobial stewardship. This systematic review aims to compare the diagnostic performance and clinical impact of mPCR versus conventional methods for diagnosing VAP in ICU settings. This review was conducted in accordance with Preferred Reporting Items for Systematic Reviews and Meta-Analyses (PRISMA) 2020 guidelines. A comprehensive search of PubMed/MEDLINE, Embase, Web of Science, Scopus, and the Cochrane Library was performed for studies published between 2020 and 2025. Eligible studies compared mPCR with conventional culture in ICU patients with suspected VAP and reported diagnostic accuracy metrics. Study quality was assessed using the QUADAS-2 tool. A qualitative synthesis was performed due to significant heterogeneity among the included studies. Fifteen studies were included. mPCR demonstrated high pooled sensitivity and specificity, with a consistently high negative predictive value (NPV) frequently approaching 100%. This high NPV provides a strong rationale for discontinuing unnecessary antibiotics when results are negative. However, positive predictive value (PPV) was more variable and often lower, reflecting the challenge of differentiating true infection from colonization. The most significant advantage of mPCR was its drastically reduced turnaround time compared to conventional culture. This rapidity facilitated earlier antibiotic modifications, including de-escalation and targeted therapy, as demonstrated in several studies. mPCR represents a significant advancement for the rapid microbiological diagnosis of VAP, offering high NPV and dramatically faster results than conventional culture. These attributes make it a powerful tool for enhancing antimicrobial stewardship in ICUs. However, its optimal use requires integration into clinical practice with careful interpretation of positive results within the context of clinical signs to distinguish infection from colonization. mPCR should be viewed as a complementary diagnostic tool that augments, rather than replaces, conventional microbiology. Limitations include potential omission of relevant studies due to database restrictions, language barriers, and paywalled articles, which may have influenced the comprehensiveness of study retrieval. Future research should focus on measuring its impact on hard clinical outcomes and conducting formal cost-effectiveness analyses.

## Introduction and background

Ventilator-associated pneumonia (VAP) remains one of the most prevalent and severe healthcare-associated infections in intensive care units (ICUs). It is clinically defined as pneumonia that develops 48 hours or more after endotracheal intubation and initiation of mechanical ventilation, characterized by new or progressive pulmonary infiltrates on chest imaging accompanied by clinical signs such as fever, purulent tracheal secretions, leukocytosis, and worsening oxygenation [1]. VAP is associated with prolonged mechanical ventilation, extended hospital stays, increased healthcare costs, and significant morbidity and mortality among critically ill patients. Timely and accurate diagnosis of VAP is therefore crucial to guide appropriate antimicrobial therapy, reduce complications, and improve patient outcomes [2].

Traditionally, the diagnosis of VAP has relied on conventional methods such as culture-based techniques and clinical criteria [3]. While microbiological cultures remain the gold standard, they are often limited by long turnaround times (TATs), reduced sensitivity due to prior antibiotic exposure, and challenges in differentiating colonization from true infection [4]. These limitations can lead to delays in treatment initiation, inappropriate antibiotic use, and the emergence of antimicrobial resistance, which poses a growing threat in ICU settings.

In recent years, Multiplex polymerase chain reaction (PCR) (mPCR) assays have emerged as promising molecular diagnostic tools for detecting respiratory pathogens directly from clinical samples [5]. mPCR offers the advantages of rapid TAT, high sensitivity, and the ability to simultaneously detect multiple bacterial and viral pathogens, as well as antimicrobial resistance genes [6]. These features may facilitate early, targeted therapy and antimicrobial stewardship in critically ill patients with suspected VAP. However, questions remain regarding the accuracy, cost-effectiveness, and clinical impact of mPCR compared with conventional diagnostic methods [7].

Several studies have compared mPCR platforms with standard microbiological cultures and clinical diagnostic criteria in the context of VAP [8,9]. While some have demonstrated superior diagnostic yield and faster results, others have raised concerns about specificity, detection of non-pathogenic colonizers, and integration into clinical decision-making. A systematic evaluation of the current evidence is therefore warranted to clarify the diagnostic performance and clinical utility of mPCR relative to conventional methods in ICU practice.

This systematic review aims to critically compare mPCR with conventional diagnostic methods for VAP in ICU settings. Specifically, it will examine diagnostic accuracy, TAT, detection of key pathogens and resistance markers, and potential implications for patient management and antimicrobial stewardship.

## Review

Methodology

Study Design

This systematic review was conducted in accordance with the Preferred Reporting Items for Systematic Reviews and Meta-Analyses (PRISMA) 2020 guidelines [10].

Eligibility Criteria

This systematic review included original research studies that compared mPCR assays with conventional diagnostic methods, such as culture-based techniques, for diagnosing VAP in ICU patients. Only studies published within the last five years were considered, in order to focus on the most recent advances and ensure clinical relevance to current ICU practice. Eligible studies were those reporting diagnostic accuracy outcomes such as sensitivity, specificity, predictive values, or TAT. Reviews, conference abstracts, editorials, case reports, and studies not conducted in ICU populations were excluded.

Information Sources and Search Strategy

A comprehensive search of electronic databases was conducted, including PubMed/MEDLINE, Embase, Web of Science, Scopus, and the Cochrane Library. The search strategy was designed using a combination of controlled vocabulary and free-text terms related to “ventilator-associated pneumonia,” “multiplex PCR,” “molecular diagnostics,” and “conventional methods.” To ensure completeness, the reference lists of all included studies and relevant reviews were screened for additional eligible studies through citation searching.

Selection Process

All records retrieved from the databases were imported into EndNote (Clarivate, Philadelphia, PA) reference management software, where duplicates were identified and removed. The remaining articles were independently screened by two reviewers, first by title and abstract, and then by full text, to determine eligibility based on the inclusion and exclusion criteria. Disagreements at any stage were resolved through discussion and consensus.

Data Collection Process

Data were extracted from each included study using a standardized data extraction form. Extracted information included study characteristics (country, year of publication, study design, and sample size), patient population (ICU type and clinical characteristics), diagnostic methods compared, reference standards used, key pathogens identified, diagnostic performance outcomes (sensitivity, specificity, positive predictive value (PPV), NPV), and TAT. To minimize errors, data extraction was performed by one reviewer and cross-checked by a second reviewer.

Study Risk of Bias Assessment

The quality and risk of bias of the included studies were evaluated using the QUADAS-2 tool [11], which is specifically designed for studies of diagnostic accuracy. The tool assesses risk across four domains: Patient Selection, Index Test, Reference Standard, and Flow and Timing. Each domain was judged as having a low, high, or unclear risk of bias. The results of the assessment are presented in tabular form.

Synthesis of Results

Given the heterogeneity in study designs, patient populations, mPCR platforms, and reference standards used across the included studies, conducting a quantitative meta-analysis was not appropriate. Pooling the results could have introduced significant bias and misrepresented the true diagnostic performance of mPCR in the clinical setting. Therefore, a qualitative synthesis was performed, with findings summarized narratively and presented in comparative tables to highlight similarities, differences, and key trends across studies.

Results

Study Selection and Characteristics

**Figure 1:**
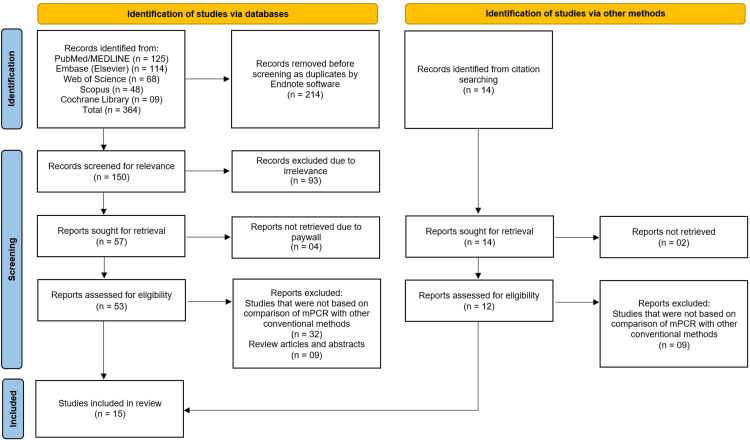
Preferred Reporting Items for Systematic Reviews and Meta-Analyses (PRISMA) flowchart

A comprehensive literature search across PubMed/MEDLINE, Embase, Web of Science, Scopus, and the Cochrane Library yielded a total of 364 records. An additional 14 records were identified through citation searching, bringing the total to 378 records. After removing 214 duplicates using EndNote software, 164 unique records remained for screening. Of these, 150 records were screened based on title and abstract, leading to the exclusion of 93 records due to irrelevance. The full texts of 57 articles were sought for retrieval; however, four reports could not be retrieved due to paywall restrictions, and two reports were not available. Consequently, 51 full-text articles were assessed for eligibility. Following full-text assessment, 41 reports were excluded: 32 did not compare mPCR with conventional methods, and nine were review articles or abstracts only (Figure 1).

A total of 15 studies [12-26] were included in this systematic review, published between 2020 and 2025. The characteristics of these studies are summarized in Table 1. The included studies were conducted across a diverse range of geographical locations, including France [12,14,18,20,24,26], the USA [15,16], the UK [21,23], Morocco [13], China [17], Belgium [19], Austria [22], and a multicenter European cohort [26]. Sample sizes varied considerably, ranging from 12 patients [17] to 1,576 patients [26], reflecting a mix of single-center and multicenter designs.

**Table 1:** Characteristics of included studies CAP - community-acquired pneumonia; ESBL-E - extended-spectrum beta-lactamase-producing *Enterobacterales*; HAP - hospital-acquired pneumonia; NR - not reported; PCR - polymerase chain reaction; VAP - ventilator-associated pneumonia

Author (Year)	Country	Study Design	Sample Size	Population (ICU type/patients)	Diagnostic Methods Compared	Reference Standard Used	Key Pathogens Detected
Contier et al., [12] (2025)	France	Retrospective, single-center study	114	Critically ill immunocompromised patients with acute respiratory failure requiring invasive ventilation	BioFire FilmArray Pneumonia Panel multiplex PCR (BFPP mPCR) vs. conventional culture (CC)	CC	Enterobacteria (51%), multi-resistant bacteria; others of low pathogenicity not included in mPCR panel
Aissaoui et al., [13] (2025)	Morocco	Multicenter observational study	210	Adult patients with pneumonia requiring invasive mechanical ventilation (CAP, HAP, VAP)	Multiplex PCR (mPCR) vs. conventional microbiological methods	Conventional microbiological methods	NR
Bay et al., [14] (2024)	France	Prospective	294 admitted; 168 mechanically ventilated; 41 episodes for diagnostic evaluation	ICU patients with ESBL-E carriage	BioFire® FilmArray® Pneumonia Plus Panel (mPCR) vs. CC	CC	ESBL-producing Enterobacterales (blaCTX-M), Carbapenem-resistant bacteria
Virk et al., [15] (2024)	USA	Single-centre, open-label, pragmatic, randomized controlled trial	1152 (modified intention-to-treat)	Hospitalized adults (≥18 years) with suspected pneumonia	BioFire FilmArray pneumonia panel vs. CC and antimicrobial susceptibility testing	CC and antimicrobial susceptibility testing	Gram-positive and Gram-negative bacteria
Pickens et al., [16] (2021)	USA	Observational, single-center	179 patients (386 BAL samples)	ICU patients with severe SARS-CoV-2 pneumonia requiring mechanical ventilation	Multiplex PCR panel vs. quantitative culture of BAL fluid	Quantitative culture of BAL fluid	Difficult-to-treat pathogens
Hou et al., [17] (2020)	China (Zhongshan Hospital, Fudan University)	Prospective observational study	12 patients (51 specimens)	ICU patients diagnosed with VAP after mechanical ventilation	PCR/ESI-MS vs. Routine Clinical Culture	Routine Clinical Culture	Common VAP pathogens (16 bacterial isolates confirmed, e.g., multiple bacterial species; 15 confirmed by paired culture, including typical VAP-causing bacteria)
Peiffer-Smadja et al., [18] (2020)	France	Prospective study	95 clinical samples from 85 patients	ICU patients with ventilated HAP or VAP; conducted in three ICUs (medical and infectious diseases ICU, surgical ICU, cardio-surgical ICU)	mPCR (Unyvero Hospitalized Pneumonia test) vs. conventional microbiological methods (culture)	CC methods	Gram-negative bacilli, Gram-positive cocci, extended-spectrum beta-lactamase producers (CTX-M), carbapenemase producers (NDM, OXA-48), Legionella
van der Schalk et al., [19] (2021)	Belgium	Diagnostic accuracy study	80 endotracheal aspirates (40 *Pseudomonas aeruginosa*-positive, 40 negative)	Mechanically ventilated ICU patients	Semi-quantitative culture, quantitative culture (ChromID *Pseudomonas aeruginosa*, blood agar), enrichment culture, O-antigen acetylase gene-based TaqMan qPCR, GeneXpert PA PCR assay	Extended gold standard (positive if detected by any of the four methods beyond semi-quantitative culture)	Pseudomonas aeruginosa
Luyt et al., [20] (2020)	France	Prospective observational study (Jan 2016 – Jan 2019)	93 BALF samples	ICU patients with suspected VAP undergoing bronchoscopy; BALF microscopy showing intracellular bacteria	Multiplex PCR (Unyvero pneumonia cartridge) vs. CC and antimicrobial susceptibility testing	Conventional microbiological culture (gold standard)	*Pseudomonas aeruginosa* (noted as key pathogen with resistance issues), plus bacteria/fungus included in the Unyvero panel (20 bacteria, one fungus, 19 resistance markers)
Enne et al., [21] (2022)	UK	Prospective observational study	652 samples	ICU patients receiving new or changed antibiotics for hospital-onset lower respiratory tract infections	BioFire FilmArray Pneumonia Panel, Unyvero Pneumonia Panel vs. Routine microbiology	Routine microbiology, Bayesian latent class analysis	Common HAP/VAP pathogens
Karolyi et al., [22] (2022)	Austria	Retrospective observational study	60	Critically ill COVID-19 patients in ICU with suspected HAP/VAP	Multiplex PCR (BioFire® Pneumonia Panel) vs. Microbiological culture	Microbiological culture of respiratory specimens	*Staphylococcus aureus*, *Klebsiella pneumoniae*, *Haemophilus influenzae*
Loughlin et al., [23] (2020)	UK	Prospective cohort (2 studies)	360 (194 tested)	Critically ill adults with suspected ventilator-associated pneumonia (non-neutropenic)	Mycological testing (BAL fluid culture, serum/BAL galactomannan, histology/microscopy)	Probable Aspergillus infection definition (clinical + radiological + mycological criteria)	*Aspergillus* spp.
Monard et al., [24] (2020)	France	Retrospective multicenter study	159 pneumonia episodes	Adult patients; mostly ICU (n = 129, 81%); HAP (n = 68), CAP (n = 54), VAP (n = 37)	Syndromic rapid multiplex PCR (rm-PCR) vs. conventional microbiological methods	CC (microbiologically documented episodes)	≥1 bacteria per episode
Razazi et al., [25] (2022)	France	Observational	95 patients (125 samples)	Mechanically ventilated ARDS patients in two ICUs (including 73 COVID-19 patients, 28 on ECMO)	Multiplex PCR (FilmArray Pneumonia Plus Panel) vs. CC	CC	NR
Rouzé et al., [26] (2021)	Europe	Multicenter retrospective cohort	1576	Adult ICU patients on invasive mechanical ventilation >48 hours; SARS-CoV-2, influenza, or no viral infection	NR	Clinical, radiological, and quantitative microbiological criteria	Gram-negative bacilli (*Pseudomonas aeruginosa,* *Enterobacter* spp., *Klebsiella* spp.)

The study designs were predominantly observational, including prospective [14,17,18,20,21] and retrospective [12,22,24,26] cohorts, with one randomized controlled trial [15]. The patient population consistently focused on critically ill adults in ICUs requiring invasive mechanical ventilation. The studies investigated various forms of pneumonia, including VAP, hospital-acquired pneumonia (HAP), and community-acquired pneumonia (CAP) in intubated patients, and pneumonia in specific subpopulations such as immunocompromised patients [12], those with ARDS [25], and patients with severe COVID-19 [16,22,26].

A variety of mPCR platforms were evaluated against conventional microbiological culture (CC) as the reference standard. The most commonly used platforms were the BioFire FilmArray Pneumonia Panel (BFPP) or its extended version (Pneumonia Plus Panel) [12,14,15,21,22,25] and the Unyvero Hospitalized Pneumonia (HPN) cartridge [18,20,21]. Other platforms included syndromic rapid mPCR tests [24], PCR/electrospray ionization mass spectrometry (PCR/ESI-MS) [17], and specific assays like the GeneXpert PA for *Pseudomonas aeruginosa* [19]. The key pathogens detected across studies included Gram-negative bacilli (e.g., *Pseudomonas aeruginosa*, *Klebsiella* spp., *Enterobacter* spp., ESBL-producing *Enterobacterales*) and Gram-positive cocci (e.g., *Staphylococcus aureus*), with some panels also detecting resistance markers and fungal pathogens [18,20,23].

Diagnostic Performance of Multiplex PCR

The diagnostic performance metrics of mPCR compared to conventional methods are detailed in Table 2. The reported sensitivity and specificity of mPCR were generally high, though variable across studies.

**Table 2:** Diagnostic performance of multiplex PCR versus conventional methods BAL, bronchoalveolar lavage; BALF, bronchoalveolar lavage fluid; CC, Conventional Culture; HPN, Hospitalized Pneumonia; NR, Not Reported; PCR, polymerase chain reaction; PTC, protected tracheal catheter

Author (Year)	Multiplex PCR Platform Used	Conventional Method Used	Sensitivity (%)	Specificity (%)	Positive Predictive Value (%)	Negative Predictive Value (%)	Turnaround Time (Hours)
Contier et al., [12] (2025)	BioFire FilmArray Pneumonia Panel (BFPP mPCR)	CC	89	83	52	98	2.5-4
Aissaoui et al., [13] (2025)	NR	NR	96.9	92	NR	NR	NR
Bay et al., [14] (2024)	BioFire® FilmArray® Pneumonia Plus Panel	Culture on respiratory samples	NR	85% concordance reported	60% (for blaCTX-M detected ESBL-E pneumonia)	100% (all blaCTX-M negative were culture negative)	NR
Virk et al., [15] (2024)	BioFire FilmArray Pneumonia Panel (bioMérieux, USA)	Standard culture + antimicrobial susceptibility testing	NR	NR	NR	NR	Median 20.4 (any antibiotic modification), 13.8 (antibiotic escalation), 20.7 (de-escalation for Gram-positive)
Pickens et al., [16] (2021)	Multiplex PCR panel	Quantitative culture of BAL fluid	NR	NR	NR	NR	NR
Hou et al., [17] (2020)	Sequential PCR coupled to electrospray ionization mass spectrometry (PCR/ESI-MS)	Routine clinical culture	14/16 VAP-confirmed isolates identified = ~87.5%	NR	NR	NR	~6
Peiffer-Smadja et al., [18] (2020)	Unyvero Hospitalized Pneumonia (HPN, Curetis)	Culture (BAL/PTC samples)	80% overall (90% for Gram-negative; 62% for Gram-positive cocci)	99%	NR	NR	Median 4.6 (IQR 4.4-5)
van der Schalk et al., [19] (2021)	GeneXpert PA PCR assay	Semi-quantitative culture	97.6	100	NR	NR	~0.9 (≈55 min)
Luyt et al., [20] (2020)	Unyvero pneumonia cartridge	BALF cultures (gold standard)	73	NR	NR	NR	4-5
Enne et al., [21] (2022)	BioFire FilmArray (bioMérieux) and Unyvero (Curetis)	Routine microbiology	50-100	87.5-99.5	NR	NR	NR
Karolyi et al., [22] (2022)	BioFire® Pneumonia Panel (PP)	Microbiological culture	NR	NR	NR	NR	NR
Loughlin et al., [23] (2020)	NR	BAL fluid culture, serum/BAL galactomannan, histology/microscopy	NR	NR	NR	NR	NR
Monard et al., [24] (2020)	Syndromic rapid multiplex PCR (rm-PCR)	Standard culture	NR	NR	NR	NR	NR
Razazi et al., [25] (2022)	FilmArray Pneumonia Plus Panel (bioMérieux, France)	Standard culture	93 (95% CI 84-100)	99 (95% CI 99-100)	68 (95% CI 54-83)	100 (95% CI 100-100)	NR
Rouzé et al., [26] (2021)	NR	Clinical, radiological, quantitative microbiology	NR	NR	NR	NR	NR

Sensitivity values ranged from 50% to 100% for detecting pathogens included in the respective panels [21]. Several studies reported high sensitivity; for instance, Aissaoui et al. reported a sensitivity of 96.9% [13], while van der Schalk et al. reported 97.6% for the specific detection of *Pseudomonas aeruginosa* using the GeneXpert PA assay [19]. Similarly, Razazi et al. reported a sensitivity of 93% (95% CI 84-100) for the FilmArray Pneumonia Plus Panel [25]. Specificity was consistently very high, often exceeding 90% and reaching 100% in some evaluations [19,25]. For example, Contier et al. reported a specificity of 83% and a notably high NPV of 98%, suggesting a strong ability to rule out infection when the test result is negative [12].

The PPV was more variable and often lower, reflecting the impact of colonization and the high sensitivity of mPCR. Contier et al. reported a PPV of 52% [12], and Razazi et al. reported a PPV of 68% (95% CI 54-83) [25], indicating that a positive mPCR result requires careful clinical correlation to distinguish true infection from colonization. Conversely, the NPV was consistently high, often approaching 100% [12,14,25], underscoring the utility of mPCR for discontinuing unnecessary antibiotics when results are negative.

A key advantage consistently demonstrated by mPCR was its significantly reduced TAT compared to conventional culture. TAT for mPCR results ranged from approximately 55 minutes [19] to six hours [17], with most platforms providing results within 2.5 to five hours [12,18,20]. In contrast, conventional culture and antimicrobial susceptibility testing typically require 48 to 72 hours, a delay that impacts timely clinical decision-making.

Impact on Antimicrobial Management

While not the primary focus of all studies, several investigations highlighted the potential impact of rapid mPCR results on antimicrobial stewardship. The drastically shortened TAT facilitated earlier adaptation of antibiotic therapy. Virk et al., in a randomized controlled trial, reported median times to any antibiotic modification of 20.4 hours, with escalation occurring at a median of 13.8 hours and de-escalation for Gram-positive organisms at 20.7 hours based on mPCR results [15].

Bay et al. focused on patients with ESBL-E carriage and found that while the PPV for ESBL-E pneumonia was 60%, the NPV was 100%; all patients with a negative mPCR for the blaCTX-M gene were culture-negative, potentially allowing for the avoidance of unnecessary carbapenem therapy [14]. Other studies similarly suggested that rapid negative results could support the early discontinuation of antibiotics, while positive results could guide earlier targeted or appropriate therapy, especially in cases where empiric coverage was inadequate [18,20,24,25].

Risk of Bias Findings

The overall risk of bias across the included studies was predominantly low, though some key concerns were noted in specific domains. The Patient Selection domain was judged as low risk for all 15 studies [12-26], indicating that the methods for selecting participants were appropriate. Similarly, the Reference Standard domain was rated low risk for all studies [12-26], confirming that conventional culture methods were applied correctly. For the Index Test, the risk of bias was low for most studies [12-14, 16-19, 22-25], though it was unclear for Virk et al. [15], Luyt et al. [20], and Rouzé et al. [26] due to insufficient information on whether the mPCR interpretation was blinded to culture results, resulting in an unclear risk in the Index Test domain. The Flow and Timing domain presented the most variability, with a high risk of bias identified in Aissaoui et al. [13], Hou et al. [17], Peiffer-Smadja et al. [18], and Enne et al. [21], often relating to inappropriate exclusions or timing issues between index and reference tests, while the risk was unclear for Luyt et al. [20] and low for the remaining studies [12,14-16,19,22-26]. Regarding applicability concerns, the Patient Selection and Reference Standard domains were of low concern for all studies [12-26]. Concerns regarding the Index Test were low for the majority [12-19,22-26], but were unclear for Luyt et al. [20], Enne et al. [21], and Rouzé et al. [26], typically because the test's application or representativeness for the review question was not fully detailed (Table 3).

**Table 3:** Risk of bias and applicability concerns summary (QUADAS-2)

Study (Year)	Risk of Bias				Applicability Concerns		
	Patient Selection	Index Test	Reference Standard	Flow and Timing	Patient Selection	Index Test	Reference Standard
Contier et al., [12] (2025)	Low risk	Low risk	Low risk	Low risk	Low concern	Low concern	Low concern
Aissaoui et al., [13] (2025)	Low risk	Low risk	Low risk	High risk	Low concern	Low concern	Low concern
Bay et al., [14] (2024)	Low risk	Low risk	Low risk	Low risk	Low concern	Low concern	Low concern
Virk et al., [15] (2024)	Low risk	Unclear risk	Low risk	Low risk	Low concern	Low concern	Low concern
Pickens et al., [16] (2021)	Low risk	Low risk	Low risk	Low risk	Low concern	Low concern	Low concern
Hou et al., [17] (2020)	Low risk	Low risk	Low risk	High risk	Low concern	Low concern	Low concern
Peiffer-Smadja et al., [18] (2020)	Low risk	Low risk	Low risk	High risk	Low concern	Low concern	Low concern
van der Schalk et al., [19] (2021)	Low risk	Low risk	Low risk	Low risk	Low concern	Low concern	Low concern
Luyt et al., [20] (2020)	Low risk	Low risk	Low risk	Unclear risk	Low concern	Unclear concern	Low concern
Enne et al., [21] (2022)	Low risk	Low risk	Low risk	High risk	Low concern	Unclear concern	Low concern
Karolyi et al., [22] (2022)	Low risk	Low risk	Low risk	Low risk	Low concern	Low concern	Low concern
Loughlin et al., [23] (2020)	Low risk	Low risk	Low risk	Low risk	Low concern	Low concern	Low concern
Monard et al., [24] (2020)	Low risk	Low risk	Low risk	Low risk	Low concern	Low concern	Low concern
Razazi et al., [25] (2022)	Low risk	Low risk	Low risk	Low risk	Low concern	Low concern	Low concern
Rouzé et al., [26] (2021)	Low risk	Unclear risk	Low risk	Low risk	Low concern	Unclear concern	Low concern

Discussion

This systematic review evaluated the diagnostic performance and clinical utility of mPCR platforms compared to conventional culture-based methods for diagnosing VAP in ICUs. The analysis of 15 studies reveals that mPCR technology offers transformative potential through dramatically reduced TATs (often under six hours compared to 48-72 hours for conventional methods) and excellent NPV, though its clinical implementation requires careful interpretation due to challenges in distinguishing colonization from true infection [12,15,17-20].

The consistently high sensitivity and specificity observed across studies, ranging from 87.5% to 100% for specific pathogens [13,19,25], confirms the technical reliability of mPCR systems for detecting target nucleic acids. The exceptional NPV, frequently approaching 100% [12,14,25], represents perhaps the most clinically significant advantage. This high NPV provides clinicians with confidence to discontinue unnecessary broad-spectrum antibiotics when mPCR results are negative, potentially reducing antimicrobial selective pressure in ICUs. However, the variable PPVs (52-68% in some studies [12, 25]) reflect the persistent challenge of differentiating true infection from respiratory colonization, a limitation also noted in previous meta-analyses by High et al. [27] and Webber et al. [28]. This diagnostic ambiguity necessitates careful correlation of mPCR results with clinical signs, biomarkers, and radiological findings, echoing recommendations from Klompas et al. [29] regarding VAP diagnosis generally.

The impact on antimicrobial stewardship emerges as a particularly promising aspect of mPCR implementation. The randomized controlled trial by Virk et al. [15] demonstrated that mPCR-guided therapy led to antibiotic modifications within a median of 20.4 hours, substantially faster than conventional methods. This finding aligns with research by Burillo et al. [30], who showed that rapid diagnostic methods can reduce time to appropriate antibiotic therapy by approximately 24 hours. The study by Bay et al. [14] provided particularly valuable insights for managing patients with ESBL-E carriage, showing that negative mPCR for blaCTX-M genes had 100% NPV for excluding ESBL-E pneumonia, potentially enabling avoidance of unnecessary carbapenem therapy. This application addresses a specific concern raised by Bassetti et al. [31] regarding carbapenem overuse in ICUs.

When contextualized within the broader literature, our findings both confirm and extend previous knowledge. The diagnostic accuracy metrics we observed are consistent with those reported in the study by Wang et al. [32], who found pooled sensitivity and specificity exceeding 90% for mPCR systems. However, our review provides several important advancements: inclusion of more recent studies through 2025, evaluation of diverse platforms (BioFire, Unyvero, PCR/ESI-MS, GeneXpert), and focus on specific challenging subpopulations, including immunocompromised hosts [12], COVID-19 patients [16,22,26], and ESBL-E carriers [14]. The detection of resistance markers directly from samples, as demonstrated by Peiffer-Smadja et al. [18] and Luyt et al. [20], represents a particular advantage over conventional methods, providing early warning of resistance mechanisms that can inform therapy days before traditional susceptibility results are available, addressing a critical need identified by Timbrook et al. [33] in their review of rapid diagnostic technologies.

Despite these advantages, several implementation challenges persist. The fixed pathogen menus of mPCR panels mean they cannot detect novel or uncommon pathogens outside their predetermined scope, as noted by Contier et al. [12], requiring continued use of conventional culture as a complementary method. The substantial upfront costs of equipment and test cartridges present economic barriers to widespread adoption, a concern also raised by Messacar et al. [34] regarding molecular diagnostic platforms generally. Furthermore, the clinical utility of mPCR depends heavily on effective antimicrobial stewardship programs to ensure rapid clinical response to diagnostic results, supporting the implementation model proposed by Banerjee et al. [35] that integrates rapid diagnostics with stewardship initiatives.

The findings from studies of specific populations offer particularly valuable insights. In immunocompromised patients [12], where infectious complications carry high mortality risks, the rapid exclusion of bacterial pneumonia through mPCR's high NPV could prevent unnecessary antibiotic exposure during vulnerable periods. For COVID-19 patients [16,22,26], who frequently experience bacterial superinfections but also receive empiric antibiotics, mPCR might help distinguish viral from bacterial pathology, addressing the diagnostic challenges described by Hughes et al. [36] during the pandemic.

Limitations

This systematic review has several limitations that should be acknowledged. The heterogeneity in study designs, patient populations, and mPCR platforms prevented quantitative meta-analysis. The QUADAS-2 assessment identified recurring methodological challenges in the "Flow and Timing" domain, reflecting the inherent difficulty of using culture, an imperfect reference standard with limited sensitivity, to validate a more sensitive molecular test. This fundamental mismatch may lead to an underestimation of mPCR's true performance characteristics. Additionally, publication bias may favor studies with positive results, and the generalizability of findings may be limited for some platforms or specific patient subgroups. Furthermore, most mPCR panels are fixed pathogen panels and cannot detect unusual or novel organisms, and they cannot reliably distinguish colonization from true infection. Economic considerations, including the high cost of mPCR and the need for integration with antimicrobial stewardship programs, may limit widespread implementation. Other limitations include exclusion of non-English studies and studies behind paywalls, variability in reported clinical outcomes, and potential biases in the included studies. Finally, most studies were conducted in high-resource ICU settings, which may reduce the applicability of findings to low-resource or diverse healthcare environments.

## Conclusions

Multiplex PCR represents a significant advancement in the rapid microbiological diagnosis of ventilator-associated pneumonia. Its dramatically reduced turnaround time and high negative predictive value offer powerful tools for enhancing antimicrobial stewardship in ICUs, though positive results should be interpreted cautiously as mPCR may detect dead bacteria, leading to false positives. However, the technology requires thoughtful implementation within a framework that acknowledges its limitations, particularly regarding differentiation between colonization and infection. mPCR should be viewed as a complementary diagnostic tool rather than a replacement for conventional methods, with its full potential realized through integration with antimicrobial stewardship programs and clinical correlation. Future research should focus on randomized trials measuring clinically important outcomes including mortality, ICU length of stay, and antimicrobial resistance rates, complemented by formal economic evaluations to establish the cost-effectiveness of these technologies in diverse healthcare settings.
